# Cetacean Intracytoplasmic Eosinophilic Globules: A Cytomorphological, Histological, Histochemical, Immunohistochemical, and Proteomic Characterization

**DOI:** 10.3390/ani13132130

**Published:** 2023-06-27

**Authors:** Antonio Fernández, Nakita Câmara, Eva Sierra, Manuel Arbelo, Yara Bernaldo de Quirós, Paul D. Jepson, Rob Deaville, Josué Díaz-Delgado, Cristian Suárez-Santana, Ayoze Castro, Julia N. Hernández, Ana Godinho

**Affiliations:** 1 Veterinary Histology and Pathology, Atlantic Center for Cetacean Research, University Institute of Animal Health and Food Safety (IUSA), Veterinary School, University of Las Palmas de Gran Canaria, Calle Transmontaña, s/n, 35416 Arucas, Canary Islands, Spain; antonio.fernandez@ulpgc.es (A.F.); eva.sierra@ulpgc.es (E.S.); manuel.arbelo@ulpgc.es (M.A.); yara.bernaldo@ulpgc.es (Y.B.d.Q.); cristian.suarez@ulpgc.es (C.S.-S.); ayoze.castro@ulpgc.es (A.C.); julia.hernandez@ulpgc.es (J.N.H.); ana_godinho@yahoo.com (A.G.); 2 The Oceanic Platform of the Canary Islands (PLOCAN), Carretera de Taliarte, s/n, 35200 Telde, Canary Islands, Spain; 3 Loro Parque Foundation, Avenida Loro Parque, s/n, 38400 Puerto de la Cruz, Canary Islands, Spain; 4 Zoological Society of London, Institute of Zoology, Regent’s Park, London NW1 4RY, UK; jepsonic.paul@gmail.com (P.D.J.); rob.deaville@ioz.ac.uk (R.D.); 5 Rua Central de Gandra, University Institute of Health Sciences (IUCS)-CESPU, 4585-116 Gandra, Portugal

**Keywords:** albumin, α1-antitrypsine, bycatch, cetacean, fibrinogen, intracytoplasmic hyaline globules, live-stranding, liver, marine mammal pathology

## Abstract

**Simple Summary:**

The presence of round to oval, single to multiple, hyaline eosinophilic globules inside the cytoplasmic of the hepatocytes of cetaceans are unknown. Therefore, this article aims to describe their occurrence and characterize their features using different laboratorial techniques. A total of 95 out of 115 cetaceans (83%) from 16 different species exhibited intracytoplasmic eosinophilic globules (IEGs) within the hepatocytes. These globules were positive for fibrinogen (FB, 97%), albumin (Alb, 85%), and α1-antitrypsine (A1AT, 53%), with the positivity for FB and A1AT correlated with live-stranding, hepatic congestion and a good nutritional status. The IEGs in 36 cetaceans that died due to bycatch were, all except one, FB-positive and A1AT-negative. The IEGs exhibited morphologic and compositional variations at the ultrastructural level, suggesting various stages of development. The proteomic analyses confirmed an association between the IEGs and acute phase proteins, suggesting a relationship between acute stress (i.e., bycatch), disease, and cellular protective mechanisms.

**Abstract:**

The nature, etiopathogenesis, and clinicopathologic relevance of the prevalent intracytoplasmic eosinophilic globules (IEGs) within hepatocytes of cetaceans are unknown. This study aims to evaluate the presence and characterize the IEGs in the hepatocytes of cetaceans using histochemical and immunohistochemical electron microscopy, Western blot, lectin histochemistry, and matrix-assisted laser desorption/ionization time-of-flight mass spectrometry techniques. A total of 95/115 (83%) animals (16 species) exhibited histologically evident intracytoplasmic round to oval, single to multiple, hyaline eosinophilic globules within the hepatocytes. These globules were largely PAS-positive, diastase resistant, and were immunopositive for fibrinogen (FB, 97%), albumin (Alb, 85%), and α1-antitrypsine (A1AT, 53%). The IEG positivity for FB and A1AT were correlated with live-stranding, hepatic congestion and a good nutritional status. The cetaceans lacking IEGs were consistently dead stranded and had poor body conditions. The IEGs in 36 bycaught cetaceans were, all except one, FB-positive and A1AT-negative. The IEGs exhibited morphologic and compositional variations at the ultrastructural level, suggesting various stages of development and/or etiopathogenesis(es). The glycocalyx analysis suggested an FB- and A1AT-glycosylation pattern variability between cetaceans and other animals. The proteomic analyses confirmed an association between the IEGs and acute phase proteins, suggesting a relationship between acute stress (i.e., bycatch), disease, and cellular protective mechanisms, allowing pathologists to correlate this morphological change using the acute hepatocytic cell response under certain stress conditions.

## 1. Introduction

Many diseases known in humans and animals are characterized by protein alterations and intracellular or extracellular aggregation [1]. The presence of intracellular inclusions (aggregates of stainable substances) [2] has been extensively described and multiple studies have investigated their nature and their relationship with disease [3,4,5,6,7,8]. For instance, intracytoplasmic globules may be identified in an α1-antitrypsine deficiency [9], Alzheimer’s disease [10], Parkison’s disease [11], amyloidosis [12], endotoxemia [13], toxicity [14], iatrogenic origin [15], endoplasmic storage diseases [6], a variety of tumor types [16,17,18], hepatectomy [19], hepatic congestion [20], congestive heart failure, and hypoxia [18]. Additionally, some authors have further speculated that intracytoplasmic clear globules appear as a postmortem phenomenon in animals that died of anoxia (or asphyxia), because of the increased venous blood pressure just before and continuing for some time after death [21]. Furthermore, such intracytoplasmic inclusions may receive different names based on their morphologic features (e.g., hyaline bodies [4,20], pale bodies [22]), nature (e.g., α1-antitripsine [A1AT] [23], fibrinogen [FB] [5,6], complement factors [5]), and discoverers (e.g., Mallory–Denk bodies [24], Lafora bodies [25]).

Protein aggregations are usually the result of structural modifications of proteins by both mutations and cellular oxidative damage [26]. In the liver, a classic example is the Mallory–Denk body seen in steatohepatitis as well as in other conditions, such as iatrogenic hepatotoxicity, cholestatic disorders and some hepatocellular neoplasms [8,27]. Furthermore, structurally normal yet overexpressed proteins may also accumulate [28]. These aggregates can also be identified as structures with poor conformation leading to chaperones such as ubiquitin (Ub) that identify them and lead to their degradation [8,29]. These accumulations of poorly formed or excess proteins usually occur in the endoplasmic reticulum and are caused by mechanisms that impede the normal transport of proteins to the Golgi apparatus [30]. Various methodologies, including histochemical (e.g., periodic acid–Schiff [PAS], Shikata, acid fuchsin) stains, immunomarkers (e.g., P62, Ub, intermediate filament proteins keratins 8 and 18), and ultrastructural analyses, have been used to characterize and discern between these inclusions [31,32]. The presence of hyaline globules in cetacean hepatocytes (hereafter referred to as intracytoplasmic eosinophilic globules [IEGs]) has been previously described [33,34]. In 1993, Kennedy et al. identified PAS-diastase-resistant (PAS-d) IEGs in two striped dolphins (*Stenella coeruleoalba*) with a morbillivirus infection [34]. These IEGs also had “pink points” (PPs) highlighted by phosphotungstic acid hematoxylin (PTAH). The electron microscopy results revealed that the IEGs were 4–8 μm in diameter and were irregularly delineated by a membrane. The authors proposed that the IEGs had a possible lysosomal origin, containing glycoproteins. The presence of morbilliviral particles was ruled out. Further, in 2004, Jaber et al. described IEGs in 33 of 135 animals representing nine different cetacean species, including odontocetes and mysticetes [35]. However, the nature, etiopathogenesis, and relevance of the prevalent IEGs within the hepatocytes remain unknown. To partially fill in this knowledge gap, this study aims to investigate the presence and characterize the nature of IEGs in the hepatocytes of cetaceans.

## 2. Materials and Methods

### 2.1. Selection Criteria, Biologic, and Epidemiologic Data

A total of 155 free-ranging cetaceans (18 species) with appropriate carcass conditions (very fresh, fresh, and mild autolysis) were employed in this study. The analyzed animals were 115 cetaceans stranded in the Canary Islands between 1992 and 2008 (including seven bycaught delphinids) and 40 harbor porpoises (*Phocoena phocoena*) stranded on the shores of the United Kingdom. The biologic data (age class, sex, body condition, carcass condition, morphometrics) and stranding conditions for each individual were recorded systematically [36,37,38]. Permission for the management of the stranded cetaceans in the Canary Islands was issued by the environmental department of the Canary Islands’ Government and the Spanish Ministry of Environment. The possession of the bodies and/or the samples collected from protected species (in this particular case, cetaceans) in England, by the aegis of the Institute of Zoology coordinated Cetacean Strandings Investigation Programme, was authorized under the Natural England Class License CL-01 (https://www.gov.uk/government/publications/licence-to-possess-plants-and-animals-for-scientific-purposes/licence-to-possess-and-transport-dead-specimens-of-annex-iv-and-iib-species-cl01#annex-b---research-establishments, accessed on 1 May 2023). No experiments were performed on live animals since our work was based on dead stranded cetaceans, including bycaught animals and some animals that had been stranded alive but died shortly after.

### 2.2. Pathologic Examinations

These animals were necropsied following the standardized protocols [36,37,38]. Representative tissue samples (random cross-sectional samples) of the main organs were collected. However, this study focused on the liver, where samples of 1 cm^3^ in size were collected from two or more regions, depending on the size of the liver and the presence/absence of gross lesions.

### 2.3. Histopathology

The tissue samples were fixed in 10% neutral buffered formalin (for a minimum of 24 h), embedded in paraffin wax, and processed routinely. The sections (3 µm) were stained with hematoxylin and eosin (H&E) for the histologic examination. The PAS (for polysaccharides [e.g., glycogen] and mucosubstances [e.g., glycoproteins, glycolipids, and mucins]) with and without diastase treatment (diastase breaks down glycogen), reticulin (for reticular fibers), Hall’s (for bilirubin), chromotrope aniline blue trichrome (CAB) (for collagen), and Perl’s Prussian blue (for iron) stains were conducted to better characterize the IEGs. A subjective grading scale (mild, moderate, marked) was applied.

### 2.4. Immunohistochemical Analysis

For the immunohistochemical (IHC) analysis, we employed primary polyclonal antibodies against A1AT (1:500, Dako, Santa Clara, CA, USA), FB (1:200, Dako), Alb (1:1000, Abcam, Cambridge, UK), and cytokeratin (CK8/18) (1:20, Euro-Diagnostica, Arnhem, The Netherlands). Briefly, the tissue sections were treated with proteinase for 7 min (A1AT, FB) or citrate for 10 min (Alb, CK8/18) and incubated for 30 min using a 10% normal swine serum before an overnight incubation with the primary antibodies. The sections were then incubated using swine anti-rabbit (A1AT, FB), donkey anti-sheep (Alb) or rabbit anti-mouse (CK 8/18) sera for 30 min at room temperature. For the visualization, the Strept ABC Complex (Dako) was applied for 1 h, followed by 3-amino-9-ethyl-carbazole (Sigma, St. Louis, MI, USA). For the positive controls, internal cases from our database were used. In the case of the negative controls, sequential sections of the positive control tissues were incubated using a nonimmune homologous serum instead of the primary antibody.

### 2.5. Ultrastructural Analysis

Transmission electron microscopy (TEM) was performed on the liver sections of eight dolphins (five species) that had IEGs labeled for FB and/or A1AT by the IHC analysis. The formalin-fixed samples were postfixed in a suspension using 2.5% glutaraldehyde in a 0.1 M phosphate buffer (pH 7.2). Further, using osmium tetroxide, they were stained with 1% uranyl acetate and embedded in epon (Epon 812, Fluka Chemie AG, Industriestrasse 25, CH 9470 Buchs, Switzerland). Semi-thin sections were stained with Toluidine blue. Then, ultra-thin sections were cut at 50 nm, contrasted using lead citrate, and observed under a Zeiss EM912 TEM. For the TEM interpretation and comparative purposes, liver samples without IEGs were used. A subjective grading scale (mild, moderate, marked) was applied.

### 2.6. Histochemistry, Western Blot, and Mass Spectrometry Analyses

A total of 23 liver samples from live-stranding, acute, and chronic disease and bycaught cases were selected. For the glycobiology analysis, formalin-fixed paraffin-embedded sections (3 µm) were incubated in a 10% bovine serum albumin for 30 min and incubated with six monoclonal primary antibodies (lectins) (Vector Laboratories, Burlingame, CA, USA), namely Sambucus nigra lectin (SNA), Phaseolus vulgaris erythroagglutinin (PHA-E), wheat germ agglutinin (WGA), Phaseolus vulgaris leucoagglutinin (PHA-L), Datura stramonium lectin (DSL), and Galanthus nivalis agglutinin (GNA) for 1 h at room temperature. The sections were washed three times for 5 min in a phosphate-buffered saline (PBS) and incubated for 45 min in the Strept ABC Complex (Vector Laboratories, Burlingame, CA, USA). Then, 3,3′-diaminobenzidine (Sigma, St. Louis, MI, USA) was applied after two washes of 5 min in the PBS. A subjective grading scale (mild, moderate, marked) was applied to describe the extension of the lesions.

For the proteomic analysis, fresh frozen liver samples were homogenized and placed in a HEPES sucrose buffer solution (985 µL) with phenylmethanesulfonyl fluoride (10 µL) and dithiothreitol (5 µL) at a low temperature, allowing for cell lysis to occur without protein denaturation. The samples were centrifuged at 7000× *g* rpm at 4 °C for 10 min, and the supernatant was recovered. The proteins were then quantified in an ELISA plate reader using the Bradford method at 595 nm absorbance. For electrophoresis, three 10% polyacrylamide gels were prepared. Two were used to perform the Western blot technique, while the other was stained with Comassi Blue for the matrix-assisted laser desorption/ionization time-of-flight mass spectrometry (MALDI-TOF) techniques.

For the Western blot analysis, gels were placed on a stirring plate and 20 volts and 90 amps were applied overnight at 4 °C for their transfer. The following day, the membrane along with the proteins were removed and stained with Ponceau Red to detect the bands and select the molecular weights of interest. After washing in distilled water and a tris-buffered saline with polysorbate 20 (TBST), the membrane was blocked with 10% milk for 1 h, and the primary antibodies (FB and A1AT) were incubated overnight. The next day, the membranes were washed using TBST and blocked with 0.5% skimmed milk for 20 min. Subsequently, the membranes were incubated with the secondary antibody (anti-rabbit, 1:3000) for 1 h. For visualization, the membranes were washed using TBST and SuperSignal^®^ (Thermo Scientific, Rockford, IL, USA) was added by stirring in the dark for 5 min.

For the MALDI-TOF analysis, the bands of greatest interest were cut out of the polyacrylamide gel once they were stained with Coomasie Blue. The selection was conducted by locating the bands with the highest protein concentration and the bands that did not appear consistently in all the samples. Subsequently, the gel was broken using ammonium bicarbonate 50 mM and acetonitrile (5%) and stirred for 15 min. The supernatant was removed, and the operation was repeated twice using ammonium bicarbonate (50 mM) and acetonitrile (50%). The supernatant was removed and immersed in 100 µL acetonitrile (100%). A SpeedVac was used for 20 min to dry the samples. DIEGsstion was achieved by adding trypsin 10 ng/µL to each sample with a volume adjustment (5 to 20 µL per sample). Next, 50 µL of a 20 Mm ammonium bicarbonate was added and the samples were incubated overnight at 37 °C. For the peptide extraction and crystallization, the samples were centrifuged lightly, and the supernatant was collected. Then, 60 µL of acetonitrile (60%) and trifluoroacetic acid (TFA, 0.1%) were added to the samples and stirred for 30 min at room temperature. The procedure was repeated twice. The supernatant was collected and dried for 2 h using the SpeedVac. Once the samples were dried, they were washed using acetonitrile (60%) and TFA (0.1%), followed by TFA (0.1%) alone. Next, the samples were aspirated using a Ziptop c18 pipette tip, which previously aspirated acetonitrile (100%) and TFA (0.1%). After aspirating the samples with cyanohydroxycinnamic acid 5 mg/mL in acetonitrile (50%) and TFA (0.1%), 1.2 µL of the solution was aspirated and applied to the plate for crystallization and the MALDI-TOF analysis. The peptide mass fingerprinting results were obtained and entered into two databases (SwissProt and nrNCBI) for the identification of the peptides and the corresponding proteins using a taxonomic selection (“other mammals”).

### 2.7. Statistical Analysis

Fisher’s exact test was performed to evaluate the potential associations between the presence of IEGs and the body condition, IEGs and congestion, IEGs and meningoencephalitides, meningoencephalitides and IEG–A1T1 positivity, meningoencephalitides and IEG–FB positivity, IEG–FB positivity and natural and anthropogenic pathologies, and IEG–A1T1 positivity and natural and anthropogenic pathologies.

## 3. Results

### 3.1. Study Population and Epidemiology of IEGs

A total of 95 of 115 (83%) animals, including 16 species, presented IEGs (Table 1).

The sex distribution was male (62), female (48), not recorded (5). The age distribution was fetus/neonate/calf (15), juvenile/subadult (26), adult (57), and not recorded (17). From the 115 animals, 48 (42%) were live stranded and 67 (58%) were dead stranded. A total of 36 animals were bycaught (UK, *n* = 29; Canary Islands, *n* = 7). The detailed biologic and epidemiologic data of these animals are recorded in Supplemental Table S1. IEGs were more prevalent in the live-stranded animals (90%) with acute liver congestion (Fisher test, *p*-value < 0.001) and in the individuals that had a good nutritional status (Fisher test, *p*-value = 0.025). No sex or age bias was noted (Fisher test, *p*-value = 0.842; and Chi-square test, *p*-value = 0.489, respectively). The bycaught animals presented a milder hepatic congestion compared to the live-stranded animals.

### 3.2. Histologic and Immunohistochemical Features of IEGs

**Figure 1:**
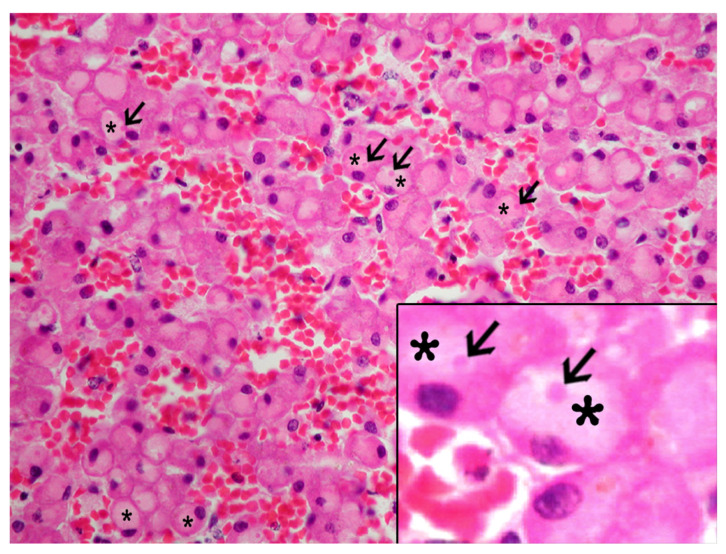
Histologic features of the intracytoplasmic eosinophilic globules (IEGs) in cetaceans. Common bottlenose dolphin, *Tursiops truncatus*. Sinusoids were variably distended; hepatocytes had intracytoplasmic IEGs (*) and “pink points” (PPs) (arrows). Hematoxylin and eosin (H&E) 40×. Inset: common bottlenose dolphin, *Tursiops truncatus.* Detail of the IEGs (*) within hepatocyte and PPs (arrows). H&E 80×.

The IEGs had varying morphologic features, ranged from 3 µm to 8 µm in diameter, were pale (occasionally noticeable as “optically empty”, bearing some resemblance to lipid macrovacuoles) to bright with thin to dense homogeneous to granular eosinophilic material that often displaced the nucleus to the periphery (signet-ring-like morphology), and had rare fibrillary structures (Figure 1). In some instances, they seemed to have a clear halo. The IEGs tended to be larger in the livers with marked congestion, typically spanning the entire cytoplasm and peripheralizing the nucleus.

**Figure 2:**
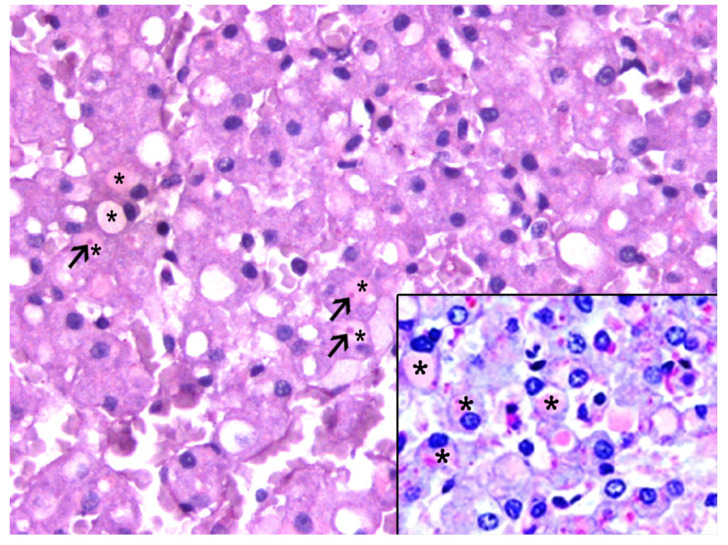
Histologic features of the intracytoplasmic eosinophilic globules (IEGs) in cetaceans. Common bottlenose dolphin, *Tursiops truncatus.* The IEGs (*) and PPs (arrows) were variably highlighted by the periodic acid–Schiff (PAS). PAS 40×. Inset: Common dolphin, *Delphinus delphis*. The IEGs (*) were largely resistant to PAS-diastase (PAS-d), clearly marked with a pinkish color. PAS-d 40×.

The IEGs were consistently yet variably highlighted by the PAS (95/95, 100%), and in 72 of 95 (76%) animals, the IEGs were PAS-d resistant (Figure 2).

The IEGs in 91 of the 95 (96%) animals had single, and rarely dual, central to peripheral bright, dense eosinophilic stippling (hereafter referred as to “pink points” [PPs]). The PPs were consistently PAS-d resistant. A moderate to marked congestion, primarily within the centrilobular areas, was observed in 90.9% of the livers with IEGs, whereas varying degrees of congestion was seen in 31.2% of the livers without IEGs.

**Figure 3:**
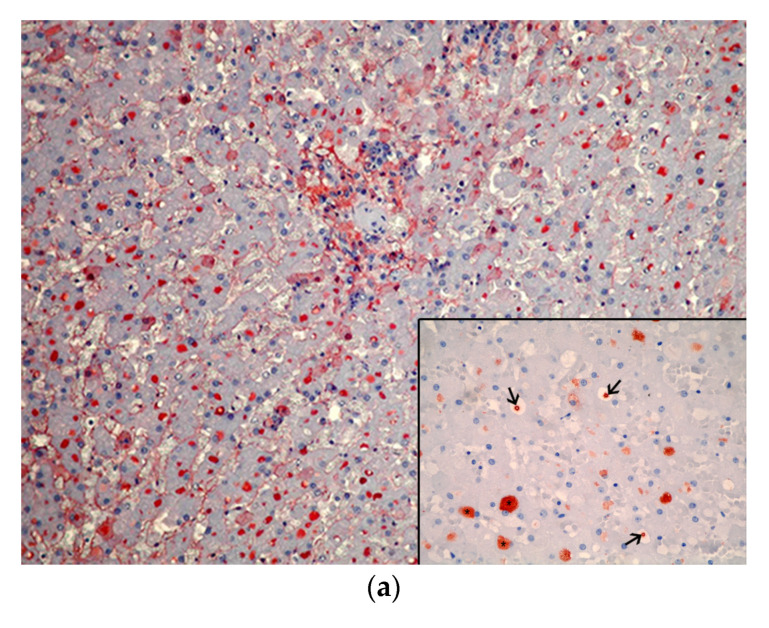

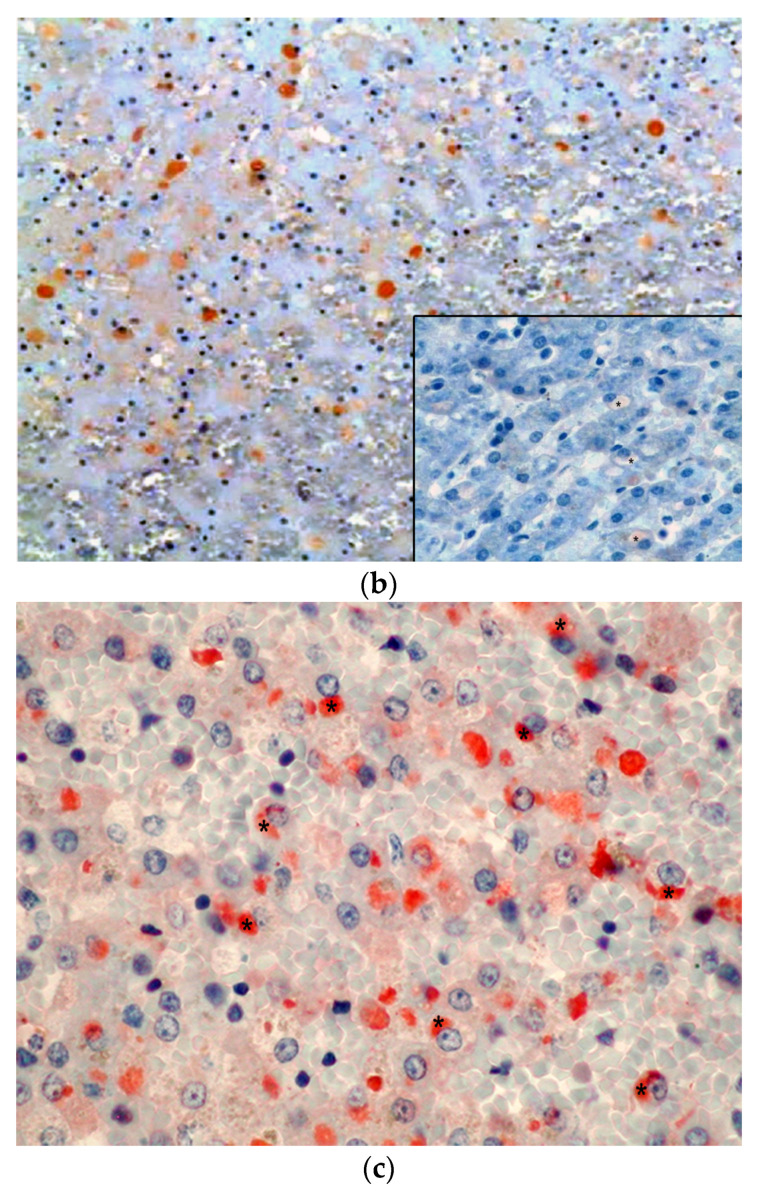
Immunohistological features of the intracytoplasmic eosinophilic globules (IEGs) in cetaceans. (**a**) Common dolphin, *Delphinus delphis*. The IEGs were labeled by an anti-fibrinogen (FB) antibody, presenting a red color. The IHC for FB 20×. Inset: common dolphin, *Delphinus delphis*. Detail of the FB-positive IEGs (*) and the PPs (arrows). The IHC for FB 40×. (**b**) Dwarf sperm whale, *Kogia sima*. The IEGs were labeled by an anti-albumin (Alb) antibody. The IHC for Alb 20×. Inset: common dolphin, *Delphinus delphis*. Detail of the Alb-positive IEGs (*). The IHC for Alb 40×. (**c**) Striped dolphin, *Stenella frontalis*. The IEGs (*) were labeled by an α-1-antritrypsin (A1AT) antibody, demonstrating a positive red color. The IHC for A1AT 40×.

The IEGs were immunopositive for FB (92/95, 97%) (Figure 3a), Alb (90/95, 90%) (Figure 3b), and A1AT (51/95, 54%) (Figure 3c) (Tables S1 and S2).

The IEGs–FB positivity was more prevalent in the animals that died due to natural causes. However, the same correlation was not found with the IEGs–A1AT positivity (Fisher test, *p*-value = 0.006 and *p*-value = 0.079, respectively). Nevertheless, the IEGs–A1AT positivity was associated with the individuals that presented meningoencephalitis (Fisher test, *p*-value = 0.024).

### 3.3. Ultrastructural Features of IEGs

**Figure 4:**
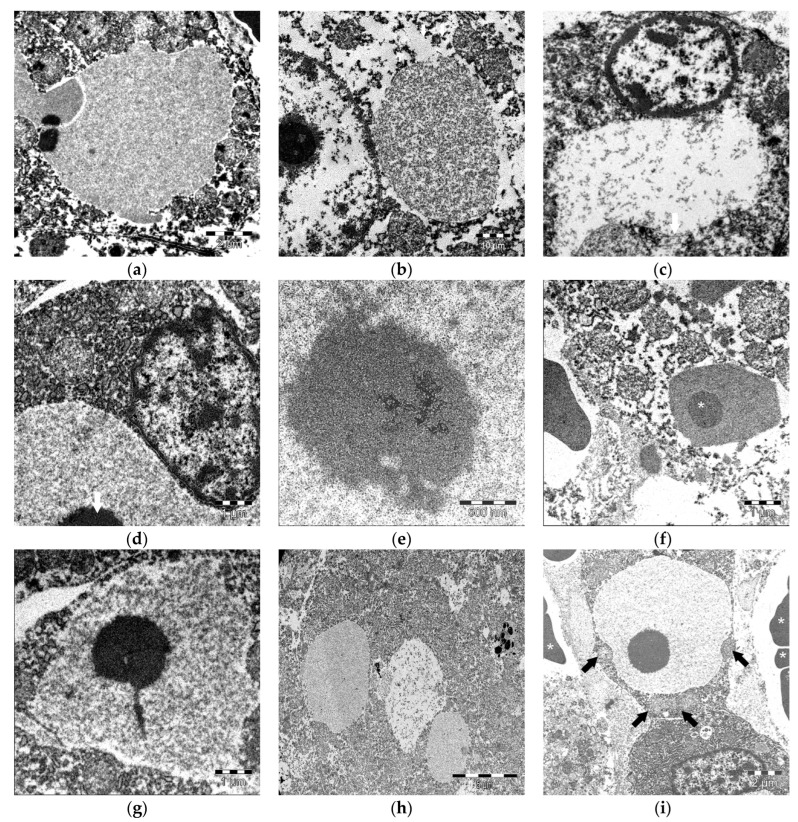
Ultrastructural features of the intracytoplasmic eosinophilic globules (IEGs) within the hepatocytes in cetaceans. Transmission electron microscopy (TEM). (**a**) Common dolphin, *Delphinus delphis*. The IEGs with a fine, moderately electrodense granular material lacking limiting membrane. 4000×. (**b**) Striped dolphin, *Stenella coeruleoalba*. Small IEGs with a granular, moderately electrodense material and a focally preserved, thin limiting membrane (arrows). 5000×. (**c**) Spotted dolphin, *Stenella frontalis*. The IEGs with a fine, scattered, mildly electrodense granular material and a focally preserved limiting membrane (arrow). 4000×. (**d**) Common bottlenose dolphin, *Tursiops truncatus*. The IEGs with a fine, moderately electrodense granular material peripheralizing the nucleus (asterisk). A fragment of a “pink point” (PP) is noted (arrow). 6300×. (**e**) Harbor porpoise, *Phocoena phocoena*. Electrodense PP lacking a limiting membrane. 20,000×. (**f**) Harbor porpoise, *Phocoena phocoena*. Large PP (asterisk) within a relatively small IEG. 4000×. (**g**) Harbor porpoise, *Phocoena phocoena*. Electrodense PP with a focal fibrillary projection within the IEG. (**h**) Harbor porpoise, *Phocoena phocoena*. Three IEGs of varying electrodensities within a hepatocyte. 6300×. (**i**) Harbor porpoise, *Phocoena phocoena*. Two PPs within an IEG; note the adjacent mitochondria (arrows). The asterisks indicate erythrocytes within sinusoidal spaces. 5000×.

Ultrastructurally, the IEGs exhibited a variable content and morphology. Some had a thin, homogenous, moderately electrodense granular material (Figure 4a), while others had a coarse, moderately electrodense granular material (Figure 4b) or a scattered thick granular electrolucent material (Figure 4c). Some IEGs were bound by a limiting membrane (Figure 4b,c). The IEGs ranged from 3 µm to 8 µm, often peripheralizing the nucleus (Figure 4d). The PPs ranged from 0.9 µm to 2.5 µm and were composed of a granular, thick electrodense material that lacked a limiting membrane (Figure 4e). The PPs often comprised a large portion of the IEGs (Figure 4f). Some of the PPs had radial projections of an identical electrodense granular material (Figure 4g). Most of the IEGs had a single PP; however, some had two. The PPs and IEGs diameters were correlated positively. In the bycaught animals, the IEGs were not morphologically dissimilar to those observed under other circumstances. Single or multiple IEGs were seen in the hepatocytes of bycaught porpoises (Figure 4h), often having a finely granular, homogeneous, and slightly electrodense material. The IEGs contours were variable. Some were lined by organelles, e.g., mitochondria, peroxisomes (Figure 4i). PPs were also detected in the bycaught animals. No additional structural abnormalities were detected in the affected hepatocytes.

### 3.4. Histochemistry, Western Blot, and Mass Spectrometry Analyses

**Figure 5:**
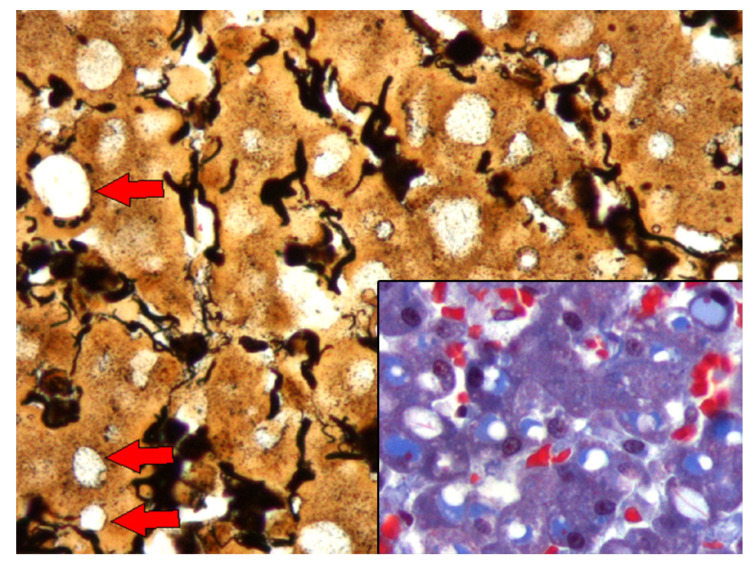
Reticulin technique. Detail of the hepatic intralobular reticulin fiber framework and adjacent hepatocytes harboring IEGs. Some IEGs had a thin peripheral stippling highlighted by a reticulin stain (arrows). Reticulin 40×. Inset: PPs (fibrillary strands in the longitudinal section) were stained red by chromotrope aniline blue trichrome (CAB). CAB 40×.

The glycobiology techniques revealed that the PPs were PAS-d resistant, had a stippling crown highlighted by a reticulin stain, and showed a red refringent staining pattern with CAB staining that was compatible with megamitochondria (Figure 5). The accumulation of glycogen within the IEGs was seen in 15 livers. The lectin histochemistry panel (Table S3) in the liver sections from three animals revealed IEGs that were consistently positive for SNA and PHA-E, and had varying staining for GNA, PHA-L, and DSA (Figure S1). The IEGs had positivity for SNA in 8/15 and PHA-E in 13/15 cases. WGA was consistently negative.

The Western blot analysis revealed specific bands for the studied proteins. Three bands corresponding to the alpha-, beta-, and gamma-chains of FB and one band corresponding to A1AT were identified (Figure S2). As expected, the anti-FB antibody identified bands of different molecular weights corresponding to the alpha-, beta-, and gamma-chains of FB and the FB-derived peptides.

A total of 19 proteins with a statistical significance were identified from 17 bands in the polyacrylamide gel (Figure S3 and Table S4). The identified proteins were serum albumin, protein disulfide-isomerase, catalase, protein disulfide isomerase-associated 3, calreticulin, carbamoyl-phosphate synthetase 1 (two isoforms), endoplasmin, formyltetrahydrofolate dehydrogenase isoform a, betaine-homocysteine methyltransferase, beta-actin, actin, alanine-glyoxylate aminotransferase, ornithine carbamoyltransferase, arginase 1, ferritin L subunit, methionine adenosyltransferase I, and glutathione S-transferase. Mitochondrial proteins were abundant, such as carbamoyl-phosphate synthetase 1, alanine-glyoxylate aminotransferase, and ornithine carbamoyltransferase, among others, as well as other proteins involved in the urea cycle, such as arginase 1, ornithine carbamoyltransferase, and carbamoyl-phosphate synthetase 1.

## 4. Discussion

This study assessed the presence of and characterized IEGs in cetaceans using histologic, immunohistochemical, ultrastructural, histochemistry and proteomic analyses. In this study, IEGs were prevalent in odontocetes and mysticetes, particularly in live-stranded animals with evidence of acute hemodynamic alterations (e.g., acute passive hepatic congestion) and a good nutritional status. The lower frequency of IEGs in animals with poor body conditions could be related to a liver protein synthesis compromise [39]. No species or sex bias was observed. However, IEGs were more prevalent in adult and subadult animals, probably due to overrepresentation. Regardless, these results suggest that animals of any age may possess etiopathogenic mechanisms that lead to IEG formation. A wide variety of cetacean species were affected and these had readily evident morphologic, biologic, and behavioral differences. Therefore, IEGs should be considered non-species-specific, which agrees with the observations in humans and some terrestrial animals [3,24,40,41]. IEGs were observed in animals subjected to stressful factors, including fatal anthropogenic interactions, e.g., bycatch, and acute and chronic disease processes.

The IEGs were similar across species and conditions. However, they presented slight morphologic and tinctorial variations, including consistent PPs. The IEGs were largely PAS-d resistant, which agreed with a previous study [34] and suggested that glycoproteins and/or glycolipids are components of IEGs [34,42]. Glycoproteins such as FB might also be non-resistant to PAS-d [5,6]. The accumulation of glycogen within the IEGs was seen in 15 livers. Such findings were frequently associated with acute and subacute hepatic injuries [43]. The IEGs tended to be larger in livers with marked congestion, typically spanning the entire cytoplasm and peripheralizing the nucleus. Hyaline, FB-positive, and A1AT-positive globules directly associated with liver congestion were described in livers from human autopsies, as well as in rats in which vacuoles were formed within the cytoplasm of the hepatocytes and metamorphosed into hyaline globules by condensation [4,19]. In a study conducted on rats, the formation of IEGs was observed within a 24 h period after the induction of hepatic congestion. After a reestablished circulation, half of the vacuoles had disappeared, while the other half underwent a metamorphosis, transforming into hyaline globules from the condensation of its content [19]. After 24 h of circulatory recovery, very few IEGs were observed, which confirmed the reversibility of the process [19]. A comparative approach would lend support to the potential reversibility for the IEGs in our study set, which is further supported by seemingly healthy cytological features based on the TEM examinations. Liver congestion or ischemia may also involve the production of other proteins, such as heat shock proteins [44].

The acute phase proteins (APPs) may be classified as positive or negative, depending on their serum concentrations during inflammation or hemostatic disturbances. The positive APPs included FB and A1AT, among others, whereas Alb was considered a negative APP [45]. In this study, the IEGs were largely immunopositive for FB and Alb (90%), and to a lesser extent for A1AT. The IEGs were consistently negative for CK8/18 and ubiquitin ruling out a compositional analogy to Mallory bodies and intracytoplasmic hyaline bodies [8,31,46,47].

FB-positive IEGs were more prevalent in congested centrilobular areas and live-stranded animals, suggesting an association with hemodynamic alterations [4,48]. The PPs were largely negative for FB; however, some showed a peripheral halo-like labeling. In hypoxic/ischemic liver disease, secondary to a reduced cardiac output, a rapid decrease in the serum FB ensued with a parallel increase in the liver FB due to a higher production of FB (and other APPs) in the hepatocytes [49]. The high prevalence of the FB-positive IGEs observed in this study and the increased FB expression recorded after a variety of liver injuries lends support to a non-specific hepatic injury response mechanism hypothesis [50].

Alb is the most abundant serum protein in mammals. It is a polypeptide and when mutated it may present glycosylated dominions [51,52]. The Alb values and consequently the albumin:globulin ratio are high in cetaceans compared to domestic animals [53]. In cachexia or profound malnutrition, protein production is impaired. In such circumstances, positive APPs such as FB and A1AT may increase, while negative APPs such as albumin may decrease [54]. The Alb-positive IEGs were more prevalent in the congested areas and included homogeneous and “optically empty” IEGs within the hepatocytes. The serum albumin is not a standout marker of liver disease in marine mammals. However, in cases of dehydration and shock [e.g., under “stranding stress response” (SSR)] [55,56,57,58,59,60], a high level of serum albumin may be detected [53]. By contrast, alpha-globulins such as haptoglobin, lipoproteins, and antitrypsin are important markers of acute inflammatory disease in some marine mammals and may be elevated in inflammatory and infectious diseases prior to clinical signs [53].

A1AT is a stress protein with direct defensive functions in the liver that can reduce cellular apoptosis in hepatic sinusoids in cases of hypothermia and hypoxia-reoxygenation [61]. Therefore, the A1TA positivity may be interpreted as a hepatocellular protection mechanism, and in many cases, coexists with the FB accumulation [62]. In humans, under normal conditions, the liver secretes 34 mg/kg/24 h of A1AT, which can be increased between three and five times in inflammatory and infectious processes [63]. In this study, 54% of the stranded cetaceans had A1AT-positive IEGs. Nearly half of them were live-stranded animals. In this study the presence the A1AT-positive IEGs was statistically correlated with meningoencephalitis. In humans, A1AT is increased in the serum and cerebrospinal fluid in cases of bacterial, but not viral, meningitis [30]. Meningoencephalitis in cetaceans is caused mainly by bacteria (*Brucella* sp.), viruses (*Morbillivirus* sp.), and parasitosis (trematodes) [36,37]. However, in these cases, virological and/or bacteriological studies have not been carried out, so the possible etiopathogenic relationship should be investigated in the future, given its possible diagnostic value. None of these cases had lesions that were suggestive of an A1AT deficiency [9,23,64,65]. A1AT-positive IEGs were common in livers of alcoholic patients [23]. Interestingly, A1AT appeared to be detectable later than FB based on lack of detection in the IEGs of bycaught porpoises (acutely dead by asphyxia).

All the bycaught animals in this study, except for one neonate, had FB-positive and A1AT-negative IEGs. Death by asphyxia in bycaught animals may take between 3 and 6 min [48]. Unlike terrestrial animals, cetaceans’ voluntary respiration may impede “reflex inspiration” (expected to occur in humans and terrestrial animals), resulting in a state of unconsciousness and death from hypoxia [66]. While pulmonary congestion, edema, and emphysema may be common in bycaught porpoises, hepatic congestion may be more variable [48,67]. In this study, the bycaught animals presented milder hepatic congestion compared to live-stranded animals. In bycaught dolphins, severe stress may result in a rapid and massive formation of IEGs. The absence of the A1AT positivity in these cases could be explained by a short course of severe stress and systemic hypoxia leading to death in fatal net entanglements [48,67]. A sudden increase in catecholamines, vasoactive factors, and capture myopathy-related damage may also play a role in these cases [48,67,68].

Either stranded off the Canary Islands or the United Kingdom, we confirmed the IEGs using transmission electron microscopy with slight morphologic variations, including number (one to two), size, electrodensity, presence or lack of limiting membranes, and adjacent organelles. The ultrastructural studies on the intracytoplasmic hyaline globules in various disease processes showed most cases to be composed of filamentous material [27]. In our study, only granular content was observed, though the state of preservation of the samples may have compromised their ultrastructure. It is known that in cases of poor A1AT conformation, intracytoplasmic globules may be surrounded by a membrane [62]. This may also be noted in FB-positive globules [69]. In this study, we observed IEGs with and without a membrane, which could suggest the existence of different IEG-developing mechanisms and, plausibly, uncharacterized globular materials. TEM was performed on the samples which were fixed in formalin, which might have resulted in certain artifacts that could have confounded the interpretation of TEM results. Future studies are warranted since it was described that 30% of the newly synthesized proteins may have been poorly assembled and could have been retained in the endoplasmic reticulum [70]. We hypothesize that altered protein maturation/folding could be a mechanism underlying the IEG formation in cetaceans.

The study of cellular glycocalyx allowed for a better understanding of cellular metabolism [71]. For instance, poorly formed A1AT requires the alteration of a mannose moiety to modify the structure of carbohydrates and to be recognized for its degradation [72]. We used lectin histochemistry to identify the carbohydrate structures since they constituted a group of proteins and glycoproteins with the ability to connect to sugar chains [73]. In cases of hypoxic and ischemic stress, there is an increase in glucose, which contributes to the synthesis of O-link β-N-acetylglucosamine (O-GlcNac) [74]. This type of glycosylation aided in the preservation of mitochondria by reducing cell death in mice [75]. On the other hand, O-GlcNac also increased thermotolerance, increasing its levels before an increase in heat shock protein (HSP) synthesis occurred [76]. This modulated the regulation of HSPs, particularly HSP90, as well as other stress-induced proteins, favoring protein stabilization and preventing their aggregation [77]. In this study, we used WGA to detect O-GlcNac, which participated in anti-stress mechanisms [78].

In this study, the IEGs were consistently positive for SNA and PHA-E, and had varying staining for GNA, PHA-L, and DSA. The known glycosylation patterns for the FB-positive hyaline globules would anticipate the positivity for SNA and the negativity for PHA-E [79]. However, we observed that the IEGSs had an SNA positivity in 8/15 and a PHA-E positivity in 13/15 cases. It was possible that the SNA-negative animals (7/15) could have expressed only the α chain of FB, based on the type of glycosylation. The IEGs were largely negative for WGA, as only one bycaught porpoise showed WGA-positive IEGs. This could be interpreted as a cellular response to a stressful situation, such as net entanglement, and affirmed an association with a stress response. As a preliminary conclusion on the glycobiology analysis results, it seems plausible that glycosylation of the different protein chains of FB and A1AT may be different from in the glycosylation in other mammals and/or that these were modified by metabolic factors. Future studies should address the sugar chain identification in FB of A1AT in cetaceans.

The etiopathogenic mechanisms that could play primary roles in the IEG development in cetaceans may be associated with (a) live-stranding-related phenomena, namely severe acute passive congestion due to body compression against the substrate and SSR [55,56,57,58,59,60], and (b) individual factors (species, age, nutritional status, health status). The body compression at beaching results in an impeded systemic venous return and systemic venous hypertension, primarily in the caudal vena cava and peritoneal viscera. This is particularly significant in the liver, which is supported by histologic alterations that are consistent with acute centrilobular (passive) congestion and may result in the accumulation of substances that cannot be excreted into the sinusoidal spaces. Under such circumstances, proteins and other molecules may leak into the space of Disse from the hepatocytes and sinusoid capillaries, as observed in rats [40]. The venous compression in rats resulted in IEGs within 3–6 h. However, the IEGs may be resolved 24 h after the subsequent vascular decompression (reperfusion). These data lend support to the reversibility of IEGs. In humans, IEGs have also been described under passive congestion in deaths by severe mechanical compression [80]. Along with other experimental studies, these highlight the relevance of hemodynamic disturbances in the IEG development, typically involving increased pressure at the caudal/inferior vena cava and subsequent hepatic congestion [19]. Interestingly, the vacuoles with a morphologic similarity to the present IEGs and the hepatocellular globules in other studies in various species were interpreted as a postmortem phenomenon in rats that were euthanized using CO_2_ in closed chambers [40]. The change has been variably reported and attributed to the postmortem effects of anoxia, inadequate exsanguination, or delay in necropsy. Two critical factors for postmortem hepatocyte vacuolation are hepatic anoxia, which increases the permeability of the hepatocytes, and high venous or intrahepatic blood pressure, which forces sinusoidal plasma into the hepatocytes to form the vacuoles [21]. With these two factors, the hepatocyte vacuoles were much larger and developed faster [21,81,82]. The venous blood pressure rose sharply during the first 5–10 min and persisted for hours after death, a time period coincident with the vacuole development in animals that die of anoxia or asphyxia [21]. In contrast, if the animals were bled immediately after death, the hepatocyte vacuoles were not observed, presumably due to the prevention of an increase in the venous or intrahepatic blood pressure [81,82]. In our study, we did not control the variable time-after-death necropsy and the fixation of the tissue. Therefore, those conclusions cannot be ratified. However, these vacuoles were not present in all the specimens and certainly in not all the routine cases.

Cetacean live strandings should be understood as potential life-threatening situations prompting physiopatholological mechanisms for the preservation of hemostasis (the basis of SSR) [55,56,57,58,59,60]. These may include peripheral vasoconstriction (leading to local or multiorgan hypoxia) to maintain a perfusion to the vital organs, such as the heart and brain [68]. If prolonged, venous congestion and arterial peripheral vasoconstriction can ensue and may lead to ischemic hypoxia in various organs, including the liver. We believe this is a major player for IEG development. Further negative factors in live-stranding events would include individual hyperthermia, which may also prompt APP production [83] or cause reperfusion injury.

Moreover, pre-existing or concomitant disease processes may influence IEG development. Along with other studies [84], this reinforces the idea that more proteins can be produced and found in the liver in stranded cetaceans that are subject to severe stressors. For instance, in septicemia, the liver increases APP production (e.g., FB and A1AT) as well as coagulation factors [85]. A lack of an A1AT expression in the FB-positive IEGs was observed in poor nutritional statuses, senility, infectious and neoplastic diseases, and in live-stranded animals. A1AT was commonly seen in animals with a variety of pathologic processes, such as septicemia, infectious meningoencephalitis, and parasitic sinusitis, which was in agreement with previous observations in natural and experimental diseases in humans and other animals [30,85].

PPs were previously described by Kennedy et al. in 1993 and Jaber et al. in 2004 [34,35]. The reports of structures comparable to PPs in humans and other animals include FB-III hyaline globules [69], type-III Mallory bodies [27], and structures observed in humans with cardiac dysfunction [86] and rats with autolysis [40]. Nevertheless, the significance of these structures is still unknown. The strong red refringence of PPs due to trichrome stain suggested megamitochondria [86,87]. However, this was ruled out by the TEM analysis. The PPs were shown to be composed of glycosylated elements and were present in the FB- and A1AT-positive IEGs. We surmised that the PPs in cetaceans were part of protein metabolism aggregation and participated in the morphologic signature of the stress response in cetaceans [88].

In the mammalian liver, 74% of the proteins are membrane proteins and only 11% correspond to cytosol proteins. When detected in a serum, it is a sign of mitochondrial damage, and consequently, cellular damage [89]. Endoplasmin (also known as HSP90) is directly related to situations of cellular stress, such as oxidative stress, and is normally associated with heat stress [90]. We detected endoplasmin using the MALDI-TOF analysis and it was likely related to live stranding and high environmental temperatures and/or individual hyperthermia. We also identified two types of disulfide isomerase protein, as well as calreticulin. The former was one of the stress proteins belonging to the endoplasmic reticulum response that acts in cases of oxidative stress [91]. These proteins belonged to a family of isomerases. A1AT was identified as one of the substrates for these enzymes [92]. Calreticulin, a soluble endoplasmic reticulum protein, acted through connections to proteins with conformation problems or that needed to be degraded, such as A1AT [92,93,94]. Both results corroborated the abundant presence of A1AT in the IEGs in stranded cetaceans and indicated that the cetacean hepatocytes may behave in a similar way to other species that exhibit an aggregation of A1TA [92,93,94]. The proteomic analysis in cetaceans remained comparatively limited [95,96,97,98]. The integration of the omics technologies, including proteomics, will be of great benefit to assess and understand the complexity and intricacy of the SSR and the signatures of disease in cetaceans. Such knowledge could advance cetacean care and conservation initiatives as well as environmental issues.

## 5. Conclusions

In summary, the combination of the histologic, immunohistochemical, glycosylation, Western blot, and proteomic analyses allowed for characterization of IEGs in stranded cetaceans. The IEG composition included APPs, primarily FB, Alb, and A1AT. The IEGs were interpreted as a potentially reversible response to stress and hepatic injury, and IEGs should be considered part of the stress response signature along with other biomarkers and pathologic findings in cetaceans. The relevant etiopathogenic mechanisms contributing to IEG development were associated with live-stranding-related phenomena, namely severe acute passive congestion and SSR, bycatch stress, and individual factors, from which the nutritional status and pre-existing diseases appear to be more important. While further research is warranted to fully characterize the composition of and assess the clinicopathologic relevance of IEGs in cetaceans, these results open new venues and contribute to a better understanding of cellular response to injury and protective phenomena in cetaceans, contributing to the advancement of integrative marine environment biomonitoring.

## Figures and Tables

**Table 1:** Cetaceans with intracytoplasmic eosinophilic globules (IEGs) within the hepatocytes and liver congestion.

Species	Stranded Cetaceans			Livers with IEGs	Liver Congestion ^1^
	Alive	Dead	Total		
*Balaenoptera physalus*	1	0	1	1/1	0/1
*Delphinus delphis*	8	10	18	15/18	15/18
*Globicephala macrorhynchus*	2	1	3	2/3	3/3
*Grampus griseus*	1	1	2	2/2	2/2
*Kogia breviceps*	0	5	5	3/5	3/5
*Kogia simus*	0	1	1	1/1	1/1
*Lagenodelphis hosei*	0	1	1	1/1	0/1
*Mesoplodon densirostris*	2	0	2	2/2	2/2
*Mesoplodon europaeus*	2	1	3	2/3	2/3
*Phocoena phocoena*	0	1	1	0/1	1/1
*Physeter macrocephalus*	0	4	4	3/4	3/4
*Pseudorca crassidens*	2	1	3	3/3	2/3
*Stenella coerulealba*	13	18	31	27/31	27/31
*Stenella longirostris*	1	2	3	0/3	3/3
*Stenella frontalis*	9	12	21	18/21	19/21
*Steno bredanensis*	1	1	2	2/2	2/2
*Tursiops truncatus*	5	7	12	12/12	12/12
*Ziphius cavirostris*	1	1	2	1/2	1/2
Total	48	67	115	95/115	98/115

^1^ Livers with moderate to marked sinusoid congestion.

## Data Availability

Data sharing is not applicable for this article.

## Supplementary Materials

The following supporting information can be downloaded at: https://www.mdpi.com/article/10.3390/ani13132130/s1, Table S1: Biologic, epidemiologic, and pathologic data of 115 cetaceans stranded in the Canary Islands included in this study; Table S2: Histochemical and immunohistochemical analysis of the intracytoplasmic eosinophilic globules (IEGs) included in this study; Table S3: Qualitative assessment of the lectin histochemistry labeling pattern, mild +, moderate ++, marked +++; Figure S1: Lectin-immunohistochemical features of intracytoplasmic eosinophilic globules (IEGs) in cetaceans. SNA, Sambucus nigra lectin; PHA-E, Phaseolus vulgaris erythroagglutinin; WGA, wheat germ agglutinin; PHA-L, Phaseolus vulgaris leucoagglutinin, DSL, Datura stramonium lectin; GNA, Galanthus nivalis agglutinin. Harbor porpoise, *Phocoena phocoena*, (a) through (e). Short-finned pilot whale, *Globicephala macrorhynchus*, (f) through (j). Spinner dolphin, *Stenella longirostris*, (k) through (o). (a) Canalicular pattern. (b) Globular pattern. (c) Canalicular pattern. (d) Labeling within Kupffer cells. (e) Globular and canalicular pattern. (f) Diffuse pattern. (g) Globular pattern. (h) Diffuse pattern. (i) Labeling within Kupffer cells. (j) Globular, canalicular and diffuse patterns. (k) Canalicular pattern. (l) Labeling along sinusoidal surface. (m) Canalicular and diffuse patterns. (n) Labeling within Kupffer cells. (o) Canalicular pattern; Figure S2: Western blot. Fibrinogen (left) and α1-antitrypsine (right); Figure S3: 10% acrylamide gel with markings of the 17 points from which the proteins were extracted; Table S4: Peptide mass fingerprinting (PMF) results.

## Abbreviations

*Alb*	Albumin
*APP*	Acute phase proteins
*A1AT*	α1-antitrypsine
*CAB*	Chromotrope aniline blue trichrome
*CK*	Cytokeratin
*DSL*	Datura stramonium lectin
*FB*	Fibrinogen
*GNA*	Galanthus nivalis agglutinin
*HSP*	Heat shock protein
*H&E*	Hematoxylin and eosin
*IHC*	Immunohistochemical
*IEGs*	Intracytoplasmic eosinophilic globules
*MALDI-TOF*	Matrix-assisted laser desorption/ionization time-of-flight mass spectrometry
*O-GlcNac*	O-link β-N-acetylglucosamine
*PAS*	Periodic acid–Schiff
*PAS-d*	Periodic acid–Schiff-diastase
*PBS*	Phosphate-buffered saline
*PHA-E*	Phaseolus vulgaris erythroagglutinin
*PHA-L*	Phaseolus vulgaris leucoagglutinin
*PP*	Pink point
*PTAH*	Phosphotungstic acid hematoxylin
*SNA*	Sambucus nigra lectin
*SSR*	Stranding stress response
*TBST*	Tris-buffered saline and polysorbate
*TEM*	Transmission electron microscopy
*TFA*	Trifluoroacetic acid
*WGA*	Wheat germ agglutinin
