# Supplementary material for: Cetacean Intracytoplasmic Eosinophilic Globules: A Cytomorphological, Histological, Histochemical, Immunohistochemical, and Proteomic Characterization
Source: Animals (Basel). 2023 Jun 27;13(13):2130. doi: 10.3390/ani13132130 (PMC10340075; doi:10.3390/ani13132130)
Supplement: Supplementary file 1 [file animals-13-02130-s001.zip › animals-2403542-supplementary.pdf]

Table S1. Biologic, epidemiologic and pathologic data of 115 cetaceans stranded in the Canary Islands included in this study.

No	Species	Sex	Age	Nutritional status	Stranding	Main pathologic findings / Cause of death	IGE (H&E)	PAS-d	A1AT	FB	ALB	CONG	PP
1	*Delphinus delphis*	ND	ND	ND	Dead	Infectious meningoencephalitis	+	+	+	+	+	+	+
2	*Physeter macrocephalus*	Female	ND	ND	Dead	Multisystemic parasitosis	+	+	+	+	ND	+	+
3	*Physeter macrocephalus*	Male	Newborn	ND	Dead	Neonatal weakness	+	+	+	+	ND	+	+
4	*Grampus griseus*	ND	ND	ND	Dead	ND	+	+	-	+	ND	+	+
5	*Lagenodelphis hosei*	ND	ND	ND	Dead	Nephritis	+	+	-	+	ND	-	+
6	*Delphinus delphis*	ND	ND	ND	Dead	Infectious meningoencephalitis	-	-	-	-	-	-	-
7	*Delphinus delphis*	ND	ND	ND	Dead	Senile	+	+	-	+	ND	-	+
8	*Ziphius cavirostris*	Female	Subadult	ND	Alive	Trauma	+	+	+	+	ND	+	-
9	*Stenella frontalis*	Male	Adult	ND	Dead	ND	+	-	-	+	+	+	+
10	*Stenella coeruleoalba*	Hembra	Adult	ND	Dead	ND	-	-	-	-	-	-	-
11	*Delphinus delphis*	Male	Adult	ND	Alive	Cerebral hemorrhage	+	+	+	+	+	+	+
12	*Delphinus delphis*	Male	ND	ND	Alive	Cerebral hemorrhage	+	+	+	+	ND	+	+
13	*Delphinus delphis*	Female	Cub	ND	Alive	Cerebral hemorrhage	+	+	+	+	+	+	+
14	*Pseudorca crassidens*	Male	Adult	ND	Alive	Infectious meningoencephalitis	+	-	-	+	+	+	+
15	*Delphinus delphis*	Female	Adult	Poor	Alive	Infectious meningoencephalitis	+	+	-	+	+	+	-
16	*Stenella frontalis*	Male	Adult	Good	Alive	ND	+	+	+	+	+	+	+
17	*Delphinus delphis*	Female	Adult	Poor	Dead	ND	+	+	-	+	+	+	+
18	*Delphinus delphis*	ND	ND	ND	Dead	ND	+	+	-	+	ND	+	+
19	*Stenella frontalis*	Female	ND	ND	Dead	Bronchopneumonia	+	-	-	+	ND	+	+
20	*Stenella coeruleoalba*	Male	Adult	Poor	Dead	Infectious meningoencephalitis	+	+	+	+	+	+	+
21	*Stenella coeruleoalba*	Hembra	Juvenil	ND	Alive	Infectious meningoencephalitis	-	-	-	-	-	+	-
22	*Stenella coeruleoalba*	Male	Juvenil	ND	Dead	Infectious meningoencephalitis	+	-	-	+	ND	+	+
23	*Tursiops truncatus*	Female	Adult	Good	Dead	Trauma	+	+	-	+	+	+	+
24	*Tursiops truncatus*	Female	Adult	Poor	Dead	Infectious myocarditis	+	+	-	+	+	+	+
25	*Stenella frontalis*	Macho	Adult	ND	Dead	Infectious adrenalitis	-	-	-	-	-	+	-
26	*Tursiops truncatus*	Female	ND	ND	Dead	Infectious meningoencephalitis	+	+	+	+	+	+	+
27	*Stenella frontalis*	Female	Cub	Moderate	Alive	ND	+	+	+	+	+	+	+
28	*Tursiops truncatus*	Female	Adult	Poor	Alive	Parasitic sinusitis	+	+	+	+	+	+	+
29	*Delphinus delphis*	Female	Adult	ND	Dead	Infectious meningoencephalitis	+	+	+	+	+	+	+
30	*Pseudorca crassidens*	Male	Newborn	ND	Dead	Neonatal weakness	+	+	+	+	+	+	+
31	*Kogia breviceps*	Male	ND	ND	Dead	Senile	+	+	+	+	+	+	+
32	*Stenella coeruleoalba*	Female	ND	ND	Dead	Infectious meningoencephalitis	+	+	+	+	+	+	+
33	*Physeter macrocephalus*	Hembra	Cub	Poor	Dead	Maternal separation	-	-	-	-	-	-	-
34	*Tursiops truncatus*	Male	ND	Poor	Alive	Parasitic meningoencephalitis	+	+	+	+	ND	-	+
35	*Delphinus delphis*	Macho	Adult	Poor	Alive	Sepsis	-	-	-	-	-	+	-
36	*Kogia breviceps*	Macho	Adult	Poor	Dead	Trauma	-	-	-	-	-	-	-
37	*Stenella frontalis*	Female	Juvenil	Good	Alive	Septicemia	+	+	+	+	+	+	+
38	*Stenella frontalis*	Female	ND	Poor	Dead	B-cell lymphoma	+	+	+	+	ND	+	+

39	*Stenella coeruleoalba*	Male	ND	Poor	Alive	Corynebacterium spp. bronchopneumonia; Systemic parasitosis	+	+	-	+	+	+	+
40	*Stenella coeruleoalba*	Male	ND	Poor	Alive	Infectious encephalitis; Protozoal dermatitis; Senile	+	-	-	+	+	+	+
41	*Steno bredanensis*	Male	Adult	Poor	Dead	Infectious meningoencephalitis; Systemic parasitosis	+	+	+	+	+	+	+
42	*Stenella frontalis*	Male	Juvenil	Good	Dead	By-caught	+	+	+	+	+	+	+
43	*Delphinus delphis*	Male	Adult	Good	Alive	T-cell lymphoma	+	+	+	+	+	+	+
44	*Ziphius cavirostris*	Hembra	SubAdult	Poor	Dead	Foreign body obstruction	-	-	-	-	-	-	-
45	*Balaenoptera physalus*	Female	Newborn	Poor	Alive	Fetal distress; Maternal-filial separation	+	-	-	+	+	-	+
46	*Tursiops truncatus*	Male	Adult	Poor	Dead	Systemic herpesvirosis	+	-	-	+	+	+	+
47	*Delphinus delphis*	Hembra	Juvenil	Poor	Dead	Systemic parasitosis	+	-	-	-	-	+	+
48	*Delphinus delphis*	Macho	Cub	Good	Dead	Trauma (fishing activity)	+	-	-	-	-	+	+
49	*Stenella frontalis*	Male	Adult	Poor	Alive	Systemic streptococcosis	+	+	+	+	+	+	+
50	*Globicephala macrorhynchus*	Macho	Cub	Poor	Dead	Maternal-filial separation	-	-	-	-	-	+	-
51	*Mesoplodon europaeus*	Female	Newborn	Poor	Alive	Fetal distress; Maternal-filial separation	+	-	-	+	-	+	+
52	*Stenella coeruleoalba*	Female	Adult	Good	Dead	Infectious pleuritis; Sepsis	+	+	+	+	+	+	+
53	*Tursiops truncatus*	Male	Cub	Good	Alive	ND	+	+	-	+	+	+	+
54	*Tursiops truncatus*	Female	Adult	Good	Alive	ND	+	+	-	+	+	+	+
55	*Stenella frontalis*	Macho	Adult	Poor	Alive	Systemic salmonellosis	-	-	-	-	-	-	-
56	*Stenella coeruleoalba*	Male	Adult	Good	Dead	Nocardia farcinica systemic infection	+	+	+	+	+	+	+
57	*Stenella coeruleoalba*	Male	Adult	Poor	Dead	Systemic parasitosis	+	+	-	+	-	+	+
58	*Stenella coeruleoalba*	Macho	Juvenil	Poor	Alive	By-caught	-	-	-	-	-	-	-
59	*Delphinus delphis*	Hembra	Adult	Poor	Dead	Systemic salmonellosis	-	-	-	-	-	-	-
60	*Stenella frontalis*	Female	Juvenil	Poor	Dead	Systemic parasitosis	+	+	+	+	+	+	+
61	*Stenella coeruleoalba*	Male	Adult	Good	Dead	Infectious meningoencephalitis	+	+	+	+	+	+	+
62	*Stenella frontalis*	Male	Cub	Poor	Dead	Maternal-filial separation	+	+	+	+	+	+	+
63	*Tursiops truncatus*	Male	SubAdult	Good	Dead	By-caught; Parasitic meningoencephalitis	+	+	+	+	+	+	+
64	*Stenella coeruleoalba*	Male	Adult	Good	Alive	Senile; Systemic parasitosis	+	+	+	+	+	+	+
65	*Tursiops truncatus*	Female	SubAdult	Good	Dead	By-caught; Parasitic bronchopneumonia	+	+	-	+	+	+	+
66	*Kogia breviceps*	Male	Adult	Good	Dead	Congestive heart failure	+	+	-	+	+	+	+
67	*Stenella frontalis*	Female	Adult	Poor	Alive	Otic nasitremiasis	+	+	-	+	ND	+	+
68	*Stenella coeruleoalba*	Male	Juvenil	Good	Alive	Infectious meningoencephalitis	+	+	+	+	+	+	+
69	*Stenella coeruleoalba*	Female	Adult	Good	Alive	Infectious meningoencephalitis	+	+	+	+	-	+	+
70	*Stenella coeruleoalba*	Male	Adult	Good	Alive	Infectious meningoencephalitis; Senile	+	+	-	+	+	+	+
71	*Stenella coeruleoalba*	Male	Adult	Good	Alive	Congestive heart failure	+	+	+	+	+	+	+
72	*Delphinus delphis*	Male	Cub	Good	Alive	Systemic parasitosis	+	+	+	+	+	+	+
73	*Kogia sima*	Male	Juvenil	Good	Dead	ND	+	+	+	+	+	+	+
74	*Mesoplodon densirostris*	Female	Adult	Poor	Alive	Senile	+	-	-	+	+	+	-
75	*Stenella frontalis*	Female	Adult	Good	Alive	Chronic renal disease	+	-	-	+	+	+	+
76	*Stenella frontalis*	Female	Adult	Good	Dead	ND	+	+	+	+	-	+	+

77	*Stenella coeruleoalba*	Male	Adult	Good	Dead	Otic nasitremiasis	+	+	+	+	ND	+	+
78	*Stenella frontalis*	Male	Adult	Poor	Dead	Otic nasitremiasis	+	+	+	+	+	+	+
79	*Mesoplodon densirostris*	Male	Adult	Poor	Alive	Senile	+	+	+	+	+	+	+
80	*Stenella coeruleoalba*	Male	Juvenil	Poor	Dead	Infectious meningoencephalitis; Foreign body obstruction	+	+	-	+	+	+	+
81	*Stenella coeruleoalba*	Male	Adult	Good	Dead	Infectious meningoencephalitis	+	+	+	+	+	+	-
82	*Delphinus delphis*	Male	Adult	Poor	Dead	Senile	+	-	-	+	+	+	+
83	*Mesoplodon europaeus*	Hembra	Juvenil	Poor	Dead	Trauma (fishing interaction)	-	-	-	-	-	-	-
84	*Stenella coeruleoalba*	Female	Juvenil	Good	Alive	Infectious meningoencephalitis	+	-	-	+	+	-	+
85	*Stenella frontalis*	Female	Adult	Good	Alive	Sepsis	+	-	-	+	+	+	+
86	*Stenella coeruleoalba*	Male	Adult	Good	Alive	Otic nasitremiasis	+	+	+	+	+	+	+
87	*Steno bredanensis*	Male	Juvenil	Poor	Alive	Systemic parasitosis	+	-	-	+	+	+	+
88	*Stenella longirostris*	Macho	Juvenil	Good	Dead	Behavioral pathology (social cohesion)	-	-	-	-	-	+	-
89	*Stenella longirostris*	Macho	Juvenil	Good	Dead	Behavioral pathology (social cohesion)	-	-	-	-	-	+	-
90	*Stenella longirostris*	Macho	Adult	Poor	Alive	Severa cachexia. Severe generalized dermatitis	-	-	-	-	-	+	-
91	*Stenella coeruleoalba*	Male	Juvenil	Good	Dead	Sepsis	+	+	+	+	+	+	+
92	*Stenella coeruleoalba*	Female	Adult	Good	Dead	Trauma	+	-	+	+	+	+	+
93	*Stenella coeruleoalba*	Female	Adult	Good	Dead	Infectious meningoencephalitis; spontaneous pleural rupture	+	+	+	+	+	+	+
94	*Stenella frontalis*	Macho	Adult	Good	Dead	Trauma (fishing interaction)	-	-	-	-	-	-	-
95	*Tursiops truncatus*	Female	SubAdult	Good	Alive	Infectious encephalitis; Otic nasitremiasis	+	+	+	+	+	+	+
96	*Stenella coeruleoalba*	Macho	SubAdult	Poor	Dead	Infectious meningoencephalitis	-	-	-	-	-	-	-
97	*Mesoplodon europaeus*	Female	SubAdult	Good	Alive	Trauma	+	-	-	+	+	+	+
98	*Globicephala macrorhynchus*	Female	Adult	Poor	Alive	Trauma (vessel collision)	+	+	-	+	+	+	+
99	*Kogia breviceps*	Hembra	Adult	Poor	Dead	Systemic parasitosis; Senile	-	-	-	-	-	-	-
100	*Phocoena phocoena*	Macho	Newborn	Poor	Dead	Neonatal suffering (meconium aspiration)	-	-	-	-	-	+	-
101	*Globicephala macrorhynchus*	Male	Cub	Good	Alive	Infectious meningoencephalitis and hepatitis	+	+	+	+	+	+	+
102	*Stenella frontalis*	Female	Adult	Good	Alive	Infectious meningoencephalitis and myocarditis; Systemic parasitosis	+	+	+	+	+	+	+
103	*Stenella coeruleoalba*	Male	Adult	Poor	Alive	Infectious encephalitis and adrenalitis; Systemic parasitosis	+	+	-	+	-	+	+
104	*Stenella frontalis*	Female	Adult	Good	Dead	Trauma (fishing interaction); Infectious meningoencephalitis and polyomyelitis	+	+	-	+	+	+	+
105	*Delphinus delphis*	Female	Adult	Poor	Alive	Viral (CeMV) and bacterial (Streptococcus phocae) coinfection; Systemic parasitosis	+	+	+	+	+	+	+
106	*Stenella coeruleoalba*	Male	Adult	Poor	Dead	Trauma	+	+	-	+	ND	+	+
107	*Stenella coeruleoalba*	Male	SubAdult	Poor	Dead	Infectious encephalitis, bronchopneumonia and adrenalitis	+	+	-	+	+	+	+
108	*Stenella frontalis*	Female	Adult	Poor	Dead	Primary uterine lymphoma with metastasis	+	+	-	+	ND	+	+

109	*Stenella coeruleoalba*	Male	Adult	Poor	Dead	Infectious cholangiohepatitis and bronchopneumonia; Systemic parasitosis	+	-	-	+	+	+	-
110	*Stenella coeruleoalba*	Male	Cub	Good	Alive	Fungal meningoencephalitis	+	+	+	+	+	+	+
111	*Pseudorca crassidens*	Male	Juvenil	Poor	Alive	Trauma	+	-	-	+	+	-	+
112	*Grampus griseus*	Male	Juvenil	Poor	Alive	Infectious meningoencephalitis	+	-	-	+	+	+	+
113	*Tursiops truncatus*	Female	SubAdult	Moderate	Dead	Infectious meningitis, myocarditis and bronchopneumonia; Systemic parasitosis	+	+	-	+	+	+	+
114	*Kogia breviceps*	Male	Adult	Poor	Dead	Trauma	+	+	+	+	+	+	+
115	*Physeter macrocephalus*	Hembra	Newborn	ND	Alive	Prematurity (dystocia, presumed; neonatal distress)	+	-	-	-	-	+	+

ND, not determined; IGE, intracytoplasmic eosinophilic globule (H&E); PAS-d, periodic acid Schiff – diastase; A1AT, α1-antitrypsin; FB, fibrinogen; ALB, albumin; CONG, hepatic congestion; PP, "pink point."

Table S2. Histochemical and immunohistochemical analysis of intracytoplasmic eosinophilic globules (IEGs) included in this study.

Species with IEGs	No cases	PAS-diastase resistance	α1-antitrypsine	Fibrinogen	Albumin	Pink points
Balaenoptera physalus	1	0/1	0/1	1/1	1/1	1/1
Delphinus delphis	15	12/15	8/15	13/15	10/15	14/15
Globicephala macrorhynchus	2	2/2	1/2	2/2	2/2	2/2
Grampus griseus	2	1/2	0/0	2/2	1/1	2/2
Kogia breviceps	3	3/3	2/3	3/3	3/3	3/3
Kogia simus	1	1/1	1/1	1/1	1/1	1/1
Lagenodelphis hosei	1	1/1	0/1	1/1	NI	1/1
Mesoplodon densirostris	2	1/2	1/2	2/2	2/2	2/2
Mesoplodon europaeus	2	0/0	0/2	2/2	1/2	2/2
Physeter macrocephalus	3	2/3	2/3	2/3	0/2	3/3
Pseudorca crassidens	3	1/3	1/3	3/3	3/3	3/3
Stenella coerulealba	27	22/27	16/27	27/27	21/24	25/27
Stenella frontalis	18	14/18	11/18	18/18	13/14	18/18
Steno bredanensis	2	1/2	1/2	2/2	2/2	2/2
Tursiops truncatus	12	11/12	5/12	12/12	11/11	12/12
Ziphius cavirostris	1	0/1	1/1	1/1	0/1	0/1
**Total**	**95**	**72/95**	**50/95**	**92/95**	**71/84**	**91/95**

NI, not identified

Table S3. Quantitative assessment of lectin-histochemistry labeling pattern, mild +, moderate ++, marked +++.

Lectin	Labeling pattern		
	I415/05	I262/06	I150/04
**WGA**	++ granular cytoplasmic labeling	+ granular cytoplasmic labeling	+ granular cytoplasmic labeling
**SNA**	++ globular labeling; +++ canalicular labeling	+++ globular labeling;<br>+++ canalicular and diffuse labeling	+++ canalicular labeling
**GNA**	+++ canalicular labeling	+++ diffuse pattern	++ canalicular and diffuse pattern
**PHA-E**	+ globular labeling	+++ globule labeling	+++ sinusoidal surface labeling
**PHA-L**	+ Kupffer cell labeling	++ Kupffer cell labeling	++ + Kupffer cell labeling
**DSA**	+++ canalicular labeling	++ diffuse labeling	++ canalicular labeling

SNA, *Sambucus nigra* lectin (recognition point: NeuAcα2,6Gal- o NeuAcα2,6GalNAc-); PHA-E, *Phaseolus vulgaris* Erythroagglutinin (recognition point: Bisecting GlcNAc); WGA, Wheat Germ Agglutinin (recognition point: N-acetyllucosamine (GlcNAc) and syalic acid); PHA-L, *Phaseolus vulgaris* Leucoagglutinin (recognition point: Galβ1,4GlcNAcβ1,6(Galβ1,4GlcNAcβ1,2) Manα-), DSL, *Datura stramonium* lectin (recognition point: (α-1,4) linked N-acetylglucosamine(GlcNAc)); GNA, *Galanthus nivalis* agglutinin (recognition point: Manα1,2Man- Manα1,6Man- Manα1,3Man-).

Table S4. Peptide Mass Fingerprint (PMF) results.

Band	Protein	Confidence (Database)
1	Serum albumin	CI >90% (SwissProt)
2	Protein disulfide-isomerase (prolyl 4-hydroxylase, beta subunit)	CI >99% (SwissProt; nrNCBI)
	Catalase	CI >99% (SwissProt; nrNCBI)
3	Protein disulfide isomerase-associated 3 (glucose regulated protein 58kD)	CI >99% (SwissProt; nrNCBI)
4	Calreticulin	CI >99% (SwissProt; nrNCBI)
	Various Aldehyde dehydrogenase, mitochondrial protein-related peptides	No statistical significance
5	Carbamoyl-phosphate synthetase 1, mitochondrial isoform 12	score 147, 100% (SwissProt, #7)
	Possible Integrin alpha-11	No statistical significance
6	carbamoyl-phosphate synthetase 1, mitochondrial isoform 33 ,	score 95, >99.9%

7	Endoplasmin / HSP90 / tumor rejection antigen gp96	score 98, >99%
8	formyltetrahydrofolate dehydrogenase isoform a variant (aldehyde-dehydrogenase 1 family, member L1)	score 80, >99%
9	Endoplasmin/HSP90/tumor rejection antigen gp96	Score 108, >99.9% (SwissProt, #10)
	Traces of formyltetrahydrofolate dehydrogenase	
10	Betaine-homocysteine methyltransferase	Score 218, 100%
11	Betaine-homocysteine methyltransferase	Score 70, >90%
	Beta-actin	Score 69, >90%
12	Actin	Score 149, 100%
13	Alanine-glyoxylate aminotransferase/Serine--pyruvate aminotransferase, mitochondrial	Score 131, 100% (SwissProt)
14	Ornithine carbamoyltransferase	Score 190, 100%
	Arginase 1	Score 117, 100% (SwissProt)
	Hepatocyte growth factor-regulated tyrosine kinase substrate	No statistical significance
15	Ferritin L subunit	Score 125, 100%
16	Methionine adenosyltransferase I, alpha/S-adenosylmethionine synthetase isoform q type-1	Score 160, 100 %
17	Glutathione S-transferase	Score 95, >99 %

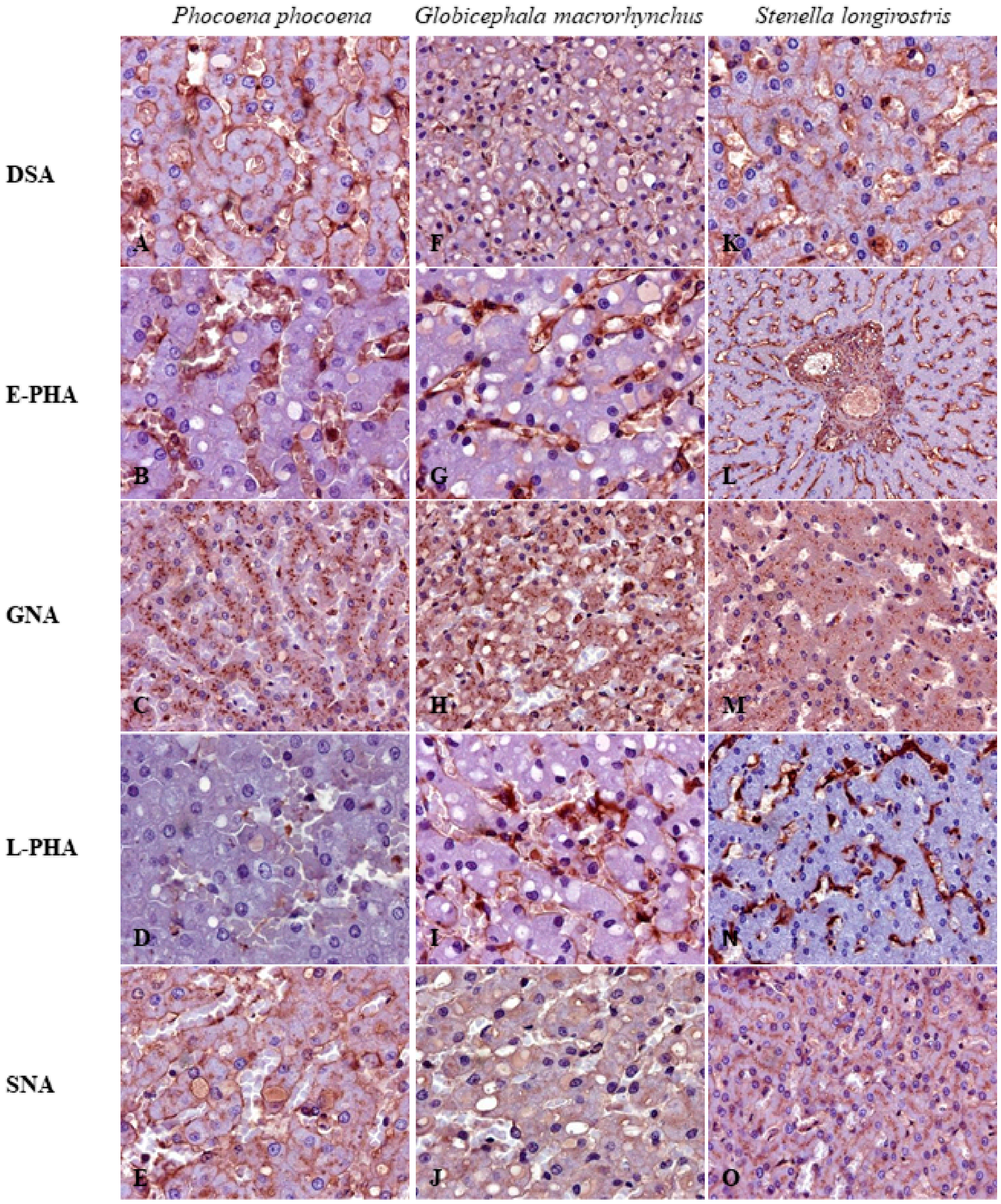

Figure S1. Lectin-immunohistochemical features of intracytoplasmic eosinophilic globules (IEGs) in cetaceans. SNA, Sambucus nigra lectin; PHA-E, Phaseolus vulgaris erythroagglutinin; WGA, wheat germ agglutinin; PHA-L, Phaseolus vulgaris leucoagglutinin, DSL, Datura stramonium lectin; GNA, Galanthus nivalis agglutinin. Harbor porpoise, *Phocoena phocoena*, (a) through (e). Short-finned pilot whale, *Globicephala macrorhynchus*, (f) through (j). Spinner dolphin, *Stenella longirostris*, (k) through (o). (a) Canalicular pattern. (b) Globular pattern. (c) Canalicular pattern. (d) Labeling within Kupffer cells. (e) Globular and canalicular pattern. (f) Diffuse pattern. (g) Globular pattern. (h) Diffuse pattern. (i) Labeling within Kupffer cells. (j) Globular, canalicular and diffuse patterns. (k) Canalicular pattern. (l) Labeling along sinusoidal surface. (m) Canalicular and diffuse patterns. (n) Labeling within Kupffer cells. (o) Canalicular pattern.

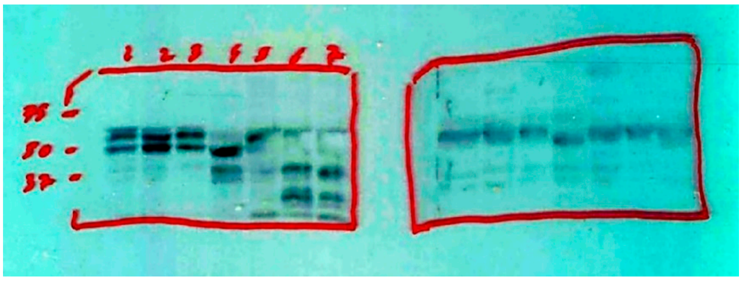

Figure S2. Western blot. Fibrinogen (left) and α1-antitrypsine (right).

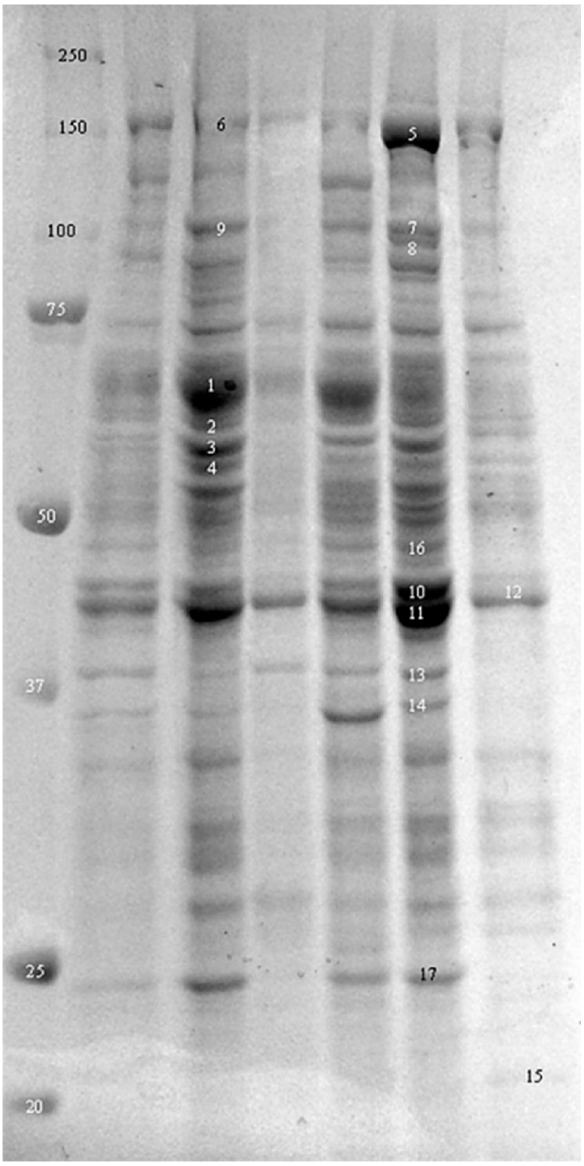

Figure S3. 10% acrylamide gel with markings of the 17 points from which the proteins were extracted.
